# Harnessing Normal and Engineered Mesenchymal Stem Cells Derived Exosomes for Cancer Therapy: Opportunity and Challenges

**DOI:** 10.3390/ijms232213974

**Published:** 2022-11-12

**Authors:** Mahdi Ahmadi, Monireh Mahmoodi, Maryam Shoaran, Fereshteh Nazari-Khanamiri, Jafar Rezaie

**Affiliations:** 1 Drug Applied Research Center, Tabriz University of Medical Sciences, Tabriz 5665665811, Iran; 2 Department of Biology, Faculty of Science, Arak University, Arak 3815688349, Iran; 3 Pediatric Health Research Center, Tabriz University of Medical Sciences, Tabriz 5665665811, Iran; 4 Solid Tumor Research Center, Cellular and Molecular Medicine Institute, Urmia University of Medical Sciences, Urmia 5714783734, Iran

**Keywords:** MSCs, mesenchymal stem cells, exosomes, tumor, drug delivery

## Abstract

There remains a vital necessity for new therapeutic approaches to combat metastatic cancers, which cause globally over 8 million deaths per year. Mesenchymal stem cells (MSCs) display aptitude as new therapeutic choices for cancer treatment. Exosomes, the most important mediator of MSCs, regulate tumor progression. The potential of harnessing exosomes from MSCs (MSCs-Exo) in cancer therapy is now being documented. MSCs-Exo can promote tumor progression by affecting tumor growth, metastasis, immunity, angiogenesis, and drug resistance. However, contradictory evidence has suggested that MSCs-Exo suppress tumors through several mechanisms. Therefore, the exact association between MSCs-Exo and tumors remains controversial. Accordingly, the applications of MSCs-Exo as novel drug delivery systems and standalone therapeutics are being extensively explored. In addition, engineering MSCs-Exo for targeting tumor cells has opened a new avenue for improving the efficiency of antitumor therapy. However, effective implementation in the clinical trials will need the establishment of standards for MSCs-Exo isolation and characterization as well as loading and engineering methods. The studies outlined in this review highlight the pivotal roles of MSCs-Exo in tumor progression and the promising potential of MSCs-Exo as therapeutic drug delivery vehicles for cancer treatment.

## 1. Introduction

**Figure 1:**
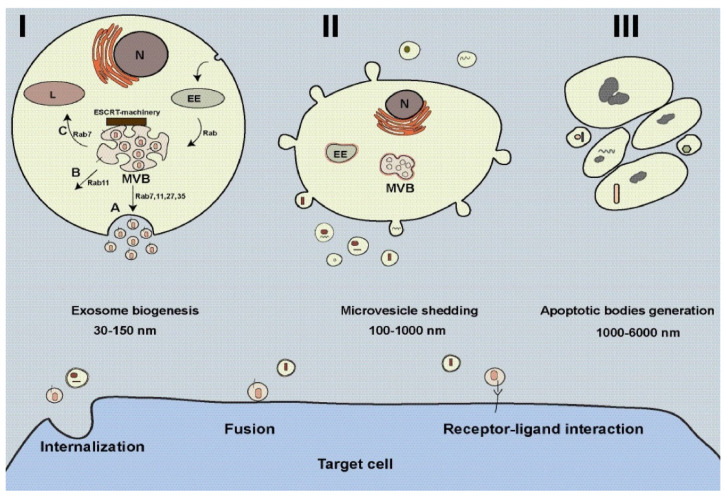
Biogenesis of extracellular vesicles (EVs) from cells. Exosome biogenesis is complex; different molecules contribute to biogenesis, trafficking, and section of exosomes (**I**). Exosomes are formed within multivesicular bodies (MVBs) located inside cells. MVBs may fuse with the plasma membrane (A), back-fuse with the plasma membrane (B), and fuse with lysosomes (C). Rabs participate in intracellular trafficking MVBs. Microvesicles are originated from the plasma membrane (**II**), and apoptotic bodies are formed from apoptotic cells (**III**). EVs can target cells in three possible ways: internalization, fusion, and receptor-ligand interaction. This figure is reused from our published article [24], under the article’s Creative Commons license. The Creative Commons CC BY license permits unrestricted use, distribution, and reproduction in any medium, provided the original work is properly cited. To view a copy of this license, visit http://creativecommons.org/licenses/by/4.0/ (accessed on 4 November 2022).

Cancer, the second leading cause of mortality worldwide, is responsible for more than 80.2 million mortality annually [1]. The tumor microenvironment (TME) is heterogeneous regarding tumor and non-tumoral cells, as well as complex interactions within cells, making tumors resistant to conventional therapies [2]. Over the last few years, despite the extensive progress in therapeutic technology, the fundamental mechanisms driving tumors remain unwell understood. Many endeavors have been performed to overcome tumor metastasis and resistance. In this regard, stem cells display hopeful results as novel therapeutic tools for cancer therapy [3]. One promising stem cell is mesenchymal stem cells (MSCs), multifunctional stem cells with the ability to differentiate into several cells and can be found in several tissues such as fat, bone marrow, dental pulp, umbilical cord, and placenta [4]. These cells contribute to tissue regeneration; however, according to previous studies, they can regulate tumor cells and immune responses [5,6]. MSCs may participate in inhibiting or/and promoting tumor progress [7,8]. MSCs may target several aspects of TME, including immune cells, endothelial cells (ECs), and fibroblasts, regulating tumor development [9,10]. MSCs can secret various soluble factors, cytokines, and immunomodulatory factors that can affect tumor cells, the phenotype of cancer-associated fibroblasts (CAFs), Ref. [9] and immune cells [11], and thereby regulate tumor progress. In addition, MSCs can be dictated by tumor-associated MSCs (TA-MSCs) and transmuted into the tumor-supporting phenotype to promote tumor growth [12]. Common knowledge is that extracellular vesicles (EVs) are the main mediators of MSCs in regenerative medicine and cancer [13]. EVs are the heterogeneous population of cell-derived vesicles that contribute to cell-to-cell communication by transferring various biomolecules like signaling molecules, RNAs, proteins, DNA strands, carbohydrates, and lipids between cells [14,15]. These vesicles release out of cells and are usually present in urine, blood, tears, saliva, cerebrospinal fluid (CSF), milk, etc. [16,17]. Once reach target cells, EVs can regulate the function, shape, and fate of cells through various signaling pathways. According to ISEV, three subclasses of EVs can be identified based on their mechanism of generation and size, known exosomes (30–150 nm), microvesicles (100–1000 nm), and apoptotic bodies (1000–6000 nm) (Figure 1 and Table 1) [14,15]. Exosomes are generated within endosomal compartments named multivesicular bodies (MVBs) inside cells via a complex mechanism involving different proteins and interactions [18,19]. Following the fusion of MVBs with the plasma membrane, exosomes are released into the extracellular matrix for targeting cells located nearby or far from (Figure 1). Therefore, exosomes play pivotal roles in normal and pathological conditions [18,19,20,21]. The main class of EVs is exosomes, which are widely studied for their function in biomedical fields and drug delivery systems. Compared with conventional nanocarriers, exosomes offer several benefits due to their distinctive physicochemical properties [22]. Due to their natural origin, exosomes show high biocompatibility, low toxicity, and low immunogenicity [22]. They also are being examined in clinical trials. Following the COVID-19 pandemic, many researchers have examined the regenerative potential of exosomes of MSCs (MSCs-Exo) in clinical trials. MSCs-Exo has been shown to regulate tumor cell growth and metastasis. Beside cell-free therapies, MSCs-Exo has received much attention in the last years regarding its application as a novel drug delivery system [23]. This review aims to summarize the current literature on the role of MSCs-Exo in cancer progression, discussing their application in drug delivery systems.

## 2. The Roles of MSCs-Exo in Tumors

**Figure 2:**
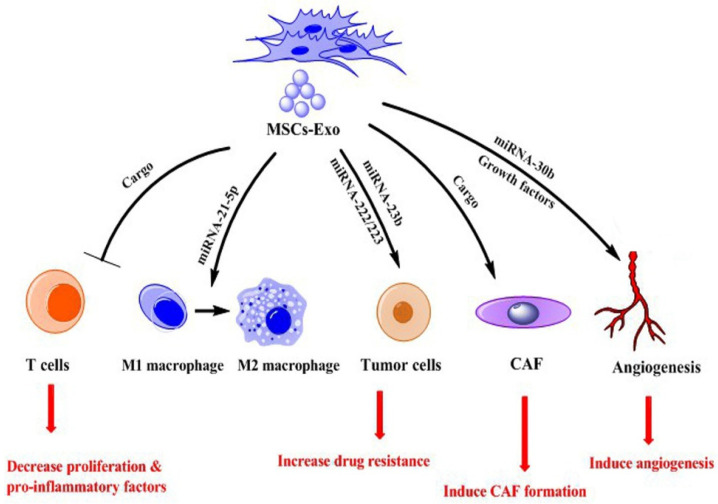
Role of exosomes from MSCs (MSCs-Exo) in promoting tumor.

TME consists of many different cells, such as endothelial cells (ECs), tumor-associated MSCs (TA-MSCs), immune cells, myeloid-derived suppressor cells (MDSCs), and tumor-associated macrophages (TAMs), creating highly complex environment [25,26]. This environment supports tumor cells in their growth and development. The main component of TME is exosomes that regulate interactions between cells resident in TME [27]. Exosomes derived from MSCs contain different miRNAs, proteins, and other biomolecules that can reprogram other cells, including tumor cells, ECs, immune cells, TAMs, MDSCs, and CAFs [7]. Previous studies report contradictory results on MSCs-Exo effects; some of them indicate these exosomes promote tumor development [28], but others show antitumor effects of them [29]. This discrepancy results may arise from the type of exosomes cargo and/or source of MSCs. For example, MSCs-Exo from bone marrow contains distinct miRNAs that could increase the proliferation of osteosarcoma cells and lung cancer [30,31]. However, He et al. showed that MSCs-Exo from a human umbilical cord source could deliver miRNA-375 to esophageal squamous cell carcinoma and decrease tumor cell progression [32]. In this section, we discuss the promoting function of MSCs-Exo in tumor development (Table 2) (Figure 2).

### 2.1. Promoting Function

#### 2.1.1. Proliferation and Metastasis

The constant growth, distribution, and metastasis of tumor cells are governed by intercellular communication between cells located in TME. Metastasis is the movement of tumor cells from the original location (primary cancer) through the circulatory system to the secondary location and the construction of new tumors (metastatic tumors) [43]. This process is a vital property of malignant tumors that is accountable for more than 90% of cancer-related death [44]. Organotypic metastasis is a feature of primary tumors to dictate secondary tumors at the metastatic location in distinct organs and comprises a chain of cell-interaction procedures recognized together as the invasion-metastasis process [44,45]. Induction of epithelial-to-mesenchymal transition (EMT) is a hallmark of aggressive tumors; therefore, cells that acquired EMT are inclined to transfer and form colonies distant from the location of origin. MSCs-Exo has been known to play roles in cancer metastasis through different signaling pathways. Zhou et al. reported that MSCs-Exo from bone marrow could increase the proliferation of tumor cells in vivo by ERK1/2 signaling pathways [40]. It was demonstrated that MSCs-Exo contains vimentin and N-cadherin molecules that promote proliferation and EMT of nasopharyngeal carcinoma by initiating the FGF19/FGFR4-dependent ERK signaling pathways [37]. Gu et al. showed that MSCs-Exo could induce EMT in gastric cancer cells by activating the AKT signaling pathway [38]. In addition, MSC-Exo from bone marrow can increase the growth and invasion of human gastric cancer cells by delivering miRNA-221 to cells in vitro [46]. Li et al. found that exosomes from MSCs contain miR-222 that could reach CRC cells, target ATF3 binding, and inhibit the activity of AKT1, increasing tumor invasion and immunosuppression of colorectal cells [47]. Lin et al. showed that MSCs-Exo from human adipose tissue promoted breast cancer progression and metastasis through activating Wnt signaling [48]. These results indicate that MSCs-Exo can support tumor progression by inducing growth and metastasis.

#### 2.1.2. Tumor Angiogenesis

Angiogenesis, raising new vessels from pre-existing vessels, is the hallmark of cancer that exosomes can regulate, participating in tumor progression [49,50]. This process is regulated by balancing pro and anti-angiogenesis factors with roles in pathological and physiological conditions [49,50]. Angiogenesis is an essential factor for tumor growth and metastasis. MSCs can release numerous growth factors and cytokines, such as VEGF, which may promote neovascularization and thus support tumor growth [51,52]. MSCs-Exo can deliver bioactive molecules to cancer cells, which induce expression of VEGF in cancer cells by activating the ERK1/2 signaling that promote tumor progression [40]. Exosomes from AT-MSCs contain platelet-derived growth factors that induce angiogenesis [39]. miRNA cargo of MSCs-Exo may participate in inducing angiogenesis. For example, exosomal miRNA-30b can promote angiogenesis in ECs [53]. In addition, MSCs-Exo has been shown to develop angiogenesis, probable through the AKT/eNOS pathway, by increasing the expression of miRNA-221-3p [54]. These findings show that MSCs-Exo contains cargo regulating angiogenesis in recipient cells.

#### 2.1.3. Tumor Immune Responses

Immune cells are vital components of the TME, interacting with other cells resident in the TME. Previous studies have shown the interaction of MSCs-Exo with immune cells such as neutrophils, T cells, B cells, and macrophages, inhibiting immune responses against tumor cells [55,56]. MSCs-Exo can suppress T-cell activity by delivering soluble factors and regulating signaling [57]. It was shown that CD30 cargo of MSCs-Exo boosted immunosuppressive effects by promoting adenosine amassing [58]. MSCs-Exo can increase the expression of anti-inflammatory molecules such as TGFB1 and IL-10 and decrease the pro-inflammatory factors IL-6, IL-1B, IL-12P40, and TNFA by prompting polymyxin-resistant SEAP expression [59]. These actions promote immunosuppression and tumor progression. Under a hypoxic condition in vivo, Ren et al. showed that MSCs-Exo carries miRNA-21-5p that can induce M2 macrophage polarization by down-regulation of PTEN, consequently increasing lung cancer growth and metastasis [35]. As known, macrophages are central constituents of the immune system. Type M2 macrophages promote tumor development by employing anti-inflammatory storms. Generally, MSCs-Exo is more capable of suppressing immune response than stimulating it.

#### 2.1.4. Tumor Drug Resistance

The main challenge in cancer management is the therapy-resistant activity of tumor cell, which cause low tumor treatment outcomes. MSCs-Exo may contribute to drug resistance [60]. For example, a study showed that MSCs-Exo from bone marrow contains PSMA3 and PSMA3-AS1 that can cause resistance to proteasome inhibitors when co-cultured with multiple myeloma cells [33]. MSCs-Exo- containing miRNA-222/223 contribute to drug resistance in breast cancer [61]. Additionally, in breast cancer cells, miRNA-23b cargo of MSCs-Exo could induce resistance to the proteasome inhibitor docetaxel [62]. Exosomes derived from MSCs induce drug (fluorouracil) resistance in gastric cancer cells by promoting mRNA levels of MRP, LRP, and MDR through the activating of calcium/calmodulin-dependent protein kinases (CaM-Ks) and the Raf/MEK/ERK cascade [63].

#### 2.1.5. TA-MSCs Derived Exosomes

While the exact function of normal MSCs on tumors remains debatable, the majority of studies on TA-MSCs suggest that they support tumor progress. Under the complex interaction within the TME, normal MSCs may acquire TA-MSCs that support tumor progression. This event can be induced by exosomes derived from tumor cells because exosomes contain pro-oncogenic factors that cause transcriptional and translational changes in normal MSCs [64]. These cells are immunosuppressive phenotypes that regulate signaling pathways that create a microenvironment favorable to tumor growth and invasion. Exosomes from TA-MSCs can regulate tumor development through different pathways and affecting on different cells in the TME [7]. For example, Yang et al. reported that TA-MSCs release exosomes that control cell migration in atypical teratoid rhabdoid tumors through the miR155/SMARCA4 pathway [65]. In breast cancer, exosomes produced by TA-MSCs transfer TGF-β, C1q, and semaphorins that can promote differentiation of myeloid cells into immunosuppressive M2-polarized macrophages by inducing PD-L1 overexpression, prompting tumor progression [36]. TA-MSCs-derived exosomes participate in the powerful immunomodulatory, which promotes tumor proliferation and invasion by producing growth factors and cytokines [66]. Moreover, exosomes from TA-MSCs deliver CCL2, CCR2, and CCL7 that can induce macrophage infiltration, increasing tumor progression [67]. These findings indicate that exosomes from TA-MSCs contribute to tumor progression.

#### 2.1.6. MSCs-Exo Alter CAFs Phenotype

In TME, soluble factors produced by cells can also modify the CAF phenotype and further support tumor progression [68]. CAFs, the main cells of TME, contribute to making pre-tumor metastatic niches with other cancer cells and provide nutrients for tumor metastasis [68]. In breast cancer, CAFs can promote tumor growth and metastasis [69]. It was suggested that exosomes secreted by TA-MSCs could induce CAFs formation [70]. Interaction within TME is complex; however, a study has shown that exosomes from gastric cancer-promoted PKM2 trigger the constant activation of the NF-κB signaling in CAFs by mediating MSC-Exo, thus disturbing inflammatory pathway and metabolic homeostasis, which offers sustained support for tumor growth [71]. The evidence of CAFs-exosomes function is little. A study indicated that exosomes from CAFs could induce chemoresistance in gastric cancer cells by delivering different miRNAs [72].

### 2.2. Tumor Suppression

**Figure 3:**
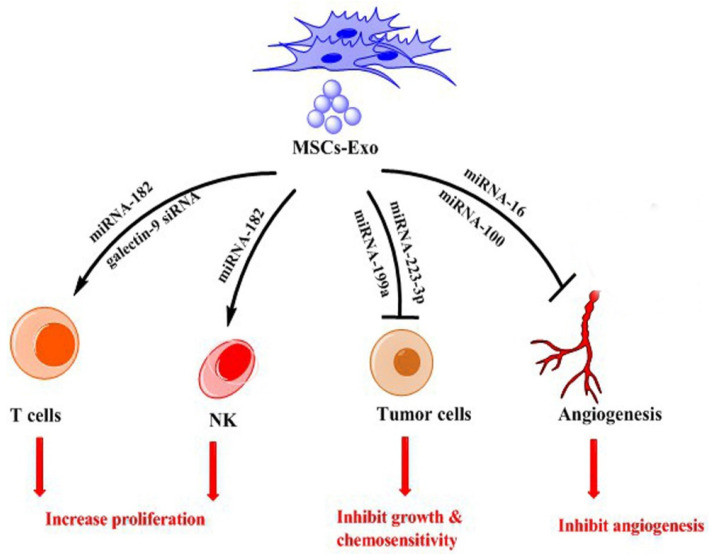
Role of exosomes from MSCs (MSCs-Exo) in inhibiting tumor.

Increasing evidence supports the key role of MSCs-Exo in the suppression of tumors via their involvement in different aspects of tumor progression (Table 3) (Figure 3).

#### 2.2.1. Proliferation and Metastasis

MSCs-Exo can suppress the proliferation and metastasis of tumors. For example, exosomes from human umbilical cord MSCs inhibit endometrial cancer cell proliferation and migration by transferring miRNA-302a and down-regulating cyclin D1 and the AKT signaling pathway [75]. Ono et al. showed that exosomes from MSCs can induce dormancy in breast cancer cells (MDA-MB-231) via transferring miRNA-23b and inhibition of MARCKS in cells, which led to the inhibition in cell cycling and migration in vitro [62]. Human umbilical cord-MSCs-Exo can reduce bladder carcinoma cell growth by inhibiting AKT phosphorylation and increasing the cleaved caspase-3 [78]. In hematological cancers, it was demonstrated that bone marrow-derived MSCs-Exo delivers miRNA-222-3p to THP-1 cells (leukemia cell), which target the IRF2 gene, consequently down-regulate the IRF2/INPP4B signaling, resulting in the inhibition of cell proliferation and the leukemia progression as well as the induction of apoptosis [73]. Yao et al. reported that bone marrow-derived-MSCs-Exo contain circ_0030167 molecules that decrease the proliferation, migration, invasion, and stemness of pancreatic tumor cells by cleaning miRNA-338-5p and consequently by targeting the Wif1/Wnt8/β-catenin signaling [79]. In glioma xenografts in the rat model, miRNA-146b cargo of MSCs-Exo decreased the growth of tumor mass; however, the detailed mechanism was not explained [80].

#### 2.2.2. Tumor Angiogenesis

MSCs can decrease angiogenesis in cancer. For example, Lee et al. showed that murine MSCs-Exo could dose-dependently decrease the expression of VEGF in breast cancer cells, suppressing angiogenesis, which may facilitate by miRNA-16 [42]. In addition, MSCs-Exo could facilitate VEGF suppression and inhibit the growth of oral squamous cell carcinoma [81] and prostate cancer cells by preventing VEGF production and NF-κB signaling [82]. Pakravan et al. demonstrated that miRNA-100 transferred by MSCs-Exo could inhibit angiogenesis and breast cancer development, through the mTOR/HIF1A/VEGF signaling pathway [74]. The roles of MSCs-Exo in angiogenesis look to be conflicting, maybe due to the type of MSCs-Exo cargo that results in different tumor regulatory properties.

#### 2.2.3. Tumor Immune Responses

The reported findings related to the role of MSCs-Exo in tumor progression show that these particles can regulate innate and adaptive immune responses. According to previous studies, comparatively few studies have examined the function of MSCs-Exo in immune stimulation, probably as MSCs-Exo mainly mediate the former rather than the latter. MSCs increase CD8+ and CD4+—T cell growth through a CCL2-related pathway [83]. Umbilical cord-MSCs-Exo can deliver miRNA-182 that can increase the death of cancer cells by increasing the proliferation of NK and T cells and by regulating the sensitivity of cancer cells to immune cells [84]. Zhou et al. engineered bone marrow-MSCs-Exo with galectin-9 siRNA and oxaliplatin and then exposed it to immune cells. Results showed that these exosomes induced antitumor immunity by suppressing Treg down-regulation, cytotoxic T lymphocyte enrolment, and macrophage polarization [85].

#### 2.2.4. Tumor Drug Resistance

MSCs-Exo may exert different impacts on drug resistance in tumor cells due to heterogeneity in tumor nature. MiR-199a-overexpressing MSCs-Exo suppressed glioma development and higher sensitivity to temozolomide by inhibiting AGAP2 expression in vitro and in vivo [86]. A recent study confirmed that AT-MSCs-Exo delivers miRNA-199a and promotes the chemosensitivity of hepatocellular carcinoma cells by the mTOR pathway [77]. The authors concluded that miRNA-199a carried by AT-MSCs-Exo might open new avenues for increasing hepatocellular carcinoma cell chemosensitivity. In a similar study, Lou et al. showed that miRNA-122 from AT-MSCs-Exo expressively improved the antitumor efficiency of sorafenib in vivo by changing chemotherapeutic drug-sensitive expression genes in hepatocellular carcinoma cells [76]. These results demonstrate that AT-MSCs-Exo can increase chemosensitivity in hepatocellular carcinoma.

## 3. MSCs-Exo as a Drug Delivery System for Cancer

**Figure 4:**
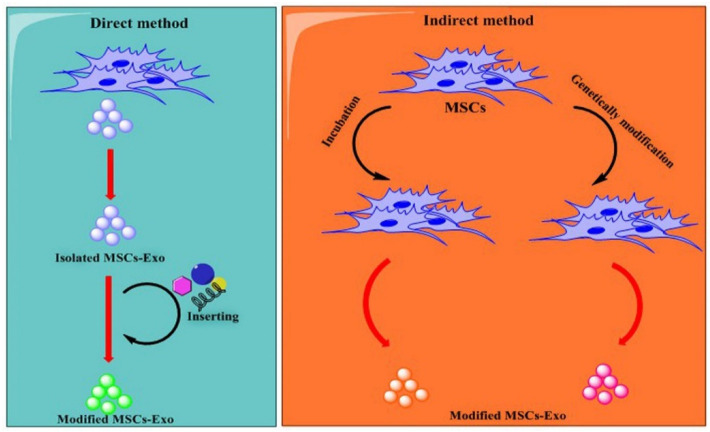
Exosomes from MSCs (MSCs-Exo) can be used as a drug delivery system. Commonly two methods are used to produce exosomes containing therapeutic agents known: the direct method and the indirect method. In direct method: MSCs-Exo are isolated and then loaded with therapeutic agents. In the indirect method: MSCs cells are modified to produce optional exosomes. Loading methods, including overexpression, transfection, electroporation, and incubation, are commonly used to load therapeutic agents.

Previous studies indicated that MSCs-exosomes could deliver therapeutic agents to tumor cells like pancreatic ductal adenocarcinoma (PDAC), CRC, hepatocellular carcinoma, breast cancer, and glioma. Generally, two methods are used to load therapeutic agents into MSCs-exosomes (i) direct method, in which therapeutic agents are directly sorted into isolated exosomes by different loading methods; (ii) indirect method, where exosomes-producing cells (e.g., MSCs) are genetically manipulated to express distinct biomolecules (miRNAs, proteins) or co-cultured with therapeutic agents in which exosomes derived from them would be contained with therapeutic agents [16,87,88] (Figure 4). Approaches currently used for the direct loading of therapeutic agents (TA) into exosomes include electroporation, incubation, extrusion, sonication, saponin, and freeze-thaw cycles. Method incubation is the most frequently used because of its simplicity; however, this method has low encapsulation efficiency [89]. Though the TA loading efficacy of electroporation is better than that of incubation, using an electric field may induce protein or RNA clump that may interrupt exosome construction or decrease the efficiency of drug delivery [90]. Compared to other approaches, sonication shows the uppermost drug loading efficiency; nevertheless, ultrasound may also interrupt the structure of exosomes and induce protein aggregation [89]. Certainly, sonication is more damaging to exosome integrity than other physical methods [91]. Related to incubation, the extrusion method produces homogeneous exosomes and improves drug delivery productivity [92]. Nevertheless, inappropriate mechanical compression can disrupt exosomal structural integrity [93]. Even though freeze-thaw cycles display capacity for mass use in drug delivery systems, multiple rapid freeze-thaw cycles may disturb the physicochemical properties of exosome membranes, and it shows less efficiency than sonication for TA loading [92,94]. The saponin method does not disturb the exosome membrane and offers high loading efficiency and constant TA release [95], but the related drug delivery efficiency desires to be enhanced. Presently, incubation and electroporation are the most commonly used approaches. Based on the outstanding properties of MSCs-Exo [96], the policy of loading MSCs-Exo with TA for tumor management using methods has been extensively engaged. For example, electroporation was used to load MSCs-Exo with doxorubicin, which could inhibit colon cancer proliferation and showed considerably greater tumor amassing than free doxorubicin [97]. Melzer et al. used the extrusion method to load paclitaxel into MSCs-Exo to treat breast cancer [98]. MSCs-Exo were constructed by modifying surfaces and loaded with superparamagnetic iron oxide nanoparticles. Proteins of cell-penetrating peptides (CPP) and TNF-α (CTNF-α)-anchored were linked to EVs containing superparamagnetic iron oxide nanoparticles. These EVs showed a targeting antitumor role and considerably suppressed tumor cell growth by inducing apoptosis by the TNFR I pathway in both in vitro and in vivo mic melanoma subcutaneous cancer models [99]. Previous studies have shown that MSCs-Exo can successfully deliver chemotherapeutic drugs to cancer cells [98,100]. In a study, MSCs-Exo were loaded with honokiol by the sonication method and exposure to cancer cells. Results showed that these particles had superior cytotoxic effects than the free honokiol [101]. Pascucci et al. used mouse MSCs-Exo to load paclitaxel (PTX) through incubation of cells with PTX and found that MSCs produced exosomes containing a high amount of PTX, which inhibited pancreatic cancer cell growth [102].

## 4. Engineering MSCs-Exo for Targeting Tumor Cells

**Figure 5:**
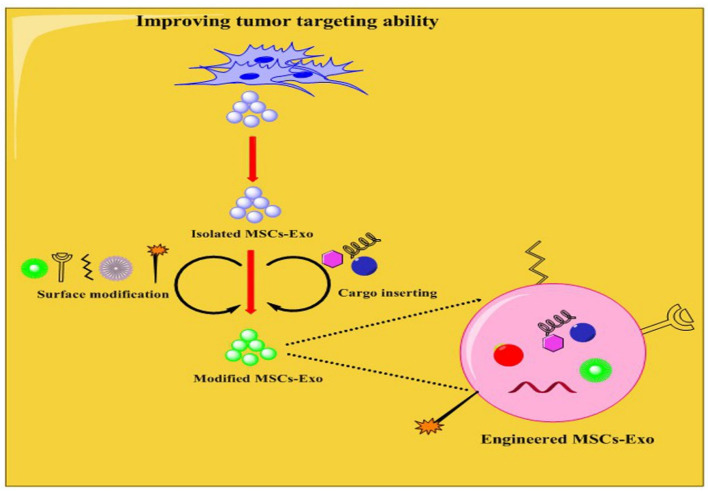
Improving the tumor-targeting ability of exosomes from MSCs (MSCs-Exo). In this theme, MSCs-Exo are modified in their content and surface to increase therapeutic efficiency. Molecules such as galectin-9 siRNA, hyaluronic acid, folate receptor, erastin, and metalloproteinases (MMPs) are used to engineer MSCs-Exo. These exosomes can be named engineered exosomes which can smartly deliver the therapeutic agent to tumor cells.

One reason for the poor therapeutic impact of chemotherapeutic drugs relates to their systemic and non-targeting effects. Even if exosomes are promising drug delivery carriers, their targeting effects need further development. Therefore, recent investigation efforts have focused on increasing the targeting capacity of exosomes to tumor cells rather than other cells, improving the efficiency of antitumor therapy (Figure 5). In this regard, engineering can increase the targeting ability of exosomes to tumor cells. The more extensively examined engineering approaches are chemical and physical modifications, like exosome surface and content modifications (for further study, see Refs. [103,104]). The surface modification comprises the application of approaches to connect protein-coding sequences or peptides into the exosome surface for improving their targeting capability [105]. Therefore, the researcher can modify/load engineered exosomes with TA and acquire smart carriers, which deliver TA into tumor cells. In a study, researchers genetically engineered MSCs to produce exosomes overexpressing miRNA-34a, which could increase the sensitivity to temozolomide, and inhibit the growth, migration, and invasion of glioblastoma cells by suppressing MYCN both in vitro and in vivo [106]. One of the approaches is the insertion of glycosyl phosphatidyl inositol (GPI) on the surface of the exosomes. GPI can attach to functional ligands like RNAs and antibodies [107,108]. It is an anchoring structure for functional ligands on the surface of the exosomes. It protects exosome surface proteins from hydrolytic degradation by proteases and directs exosomes to tumor cells. Another approach is engineering targeting peptide-Lamp2b fusion proteins for including a glycosylation motif at various sites, and these glycosylation-stabilized peptides improve the targeting ability of exosomes to tumor cells such as neuroblastoma cells [108]. In addition, the researcher introduced a method known as click chemistry, by which the ligands are attached to the exosome surface by covalent modification [109]. The benefits of chemical conjugation comprise compatibility and good chemical reaction speed; nevertheless, many factors, including pressure, temperature, and osmotic pressure, need to be carefully controlled during the modification procedure to prevent exosome rupture [109]. Therefore, these chemical approaches are highly hopeful but operationally multifaceted and need further investigation. Tian et al. conjugated the c(RGDyK) peptide to the surface of the exosomes by bio-orthogonal chemistry and formed the engineered c(RGDyK)-conjugated exosomes (cRGD-Exo), which successfully targeted injury areas of the brain in cerebral ischemia model [110]. Researchers have used a non-covalent modification to insert specific ligands or receptors on exosome surfaces [109]. For example, PEGylated liposomes were inserted into the surface of exosomes using hydrophobic interactions that considerably extended the circulation time of the exosomes and enhanced their targeting ability to murine neuroblastoma cells [111]. The targeting ability of exosomes may also be enhanced by loading targeted TAs, such as RNAs, viruses, and proteins, into the exosomes. For example, surface-modified MSCs-Exo loaded with galectin-9 siRNA showed considerably improved tumor-targeting efficacy and increased apoptosis in a pancreatic cancer model [85]. In another study, MSCs cells were modified to yield exosomes enriched with a new CFTR Zinc Finger Protein fusion and transcriptional activation elements to affect the CFTR promoter and stimulate transcription [112]. Another approach is targeting molecules highly expressed in tumor cells. For example, hyaluronic acid (HA), highly expressed in some malignancies, can target tumor cells. For example, Vogus et al. linked Hyaluronidase (HYAL) to MSCs-Exo containing gemcitabine and doxorubicin to form MSCs-Exo-HYAL. Authors reported that MSCs-Exo-HYAL targeted triple-negative breast cancer cells and significantly reduced tumor growth [113]. In addition, the folate receptor (FR), a glycoprotein, is anchored to the cell membrane by GPI. Folate is overexpressed in many tumor cells; however, its expression in normal cells is low [114]. Therefore, folate may be used as a targeting ligand for drug delivery. In this scenario, the enrichment of exosomes with FR or folate can serve as a smart carrier for finding and targeting tumor cells [115]. In a study, authors formed engineered exosomes containing FA from human umbilical cord MSCs and loaded them with erastin. These exosomes could successfully target triple-negative breast cancer cells and inhibit tumor proliferation [116]. Most recently, Feng et al. constructed Exo-PH20-FA by inserting FA into exosomes by genetic modification, which increased the efficacy of antitumor drug delivery [117]. Metalloproteinases (MMPs) also have the potential for application in tumor targeting. It was demonstrated that envelope-type mesoporous silica nanoparticles loaded with MMP substrate peptides can pretentiously target MMP-rich hepatocellular carcinoma cells [118]. Thus, overexpressed antigens or receptors on tumor cells are promising points for researchers to benefit from targeting tumor cells within tissues. Researchers can use this feature to load exosomes with drugs and direct them into tumors [103,119]. Of note, in this section, we focused on MSCs-Exo; however other exosomes or cells may be modified by targeting other molecules. This field is in its infancy, and further studies must confirm these findings for application in the clinic.

## 5. Opportunity and Challenges

Exosomes show many promising properties against artificial nanocarriers. For example, exosomes are natural vesicles produced by cells, have lower immunogenicity and long circulating half-life, superior biocompatibility, evading phagocytosis, greater modifying potential, and better targeting ability [120,121]. In addition, exosomes can cross many biological barriers and deeply penetrate tissue [122,123]. The application of exosomes as vehicles for drug delivery is presently the focus of deep research. As a drug delivery tool, MSCs-Exo has common exosomal individualities and distinctive advantages. It is important to mention that MSCs produce more exosomes compared with other cells [96] and their exosomes have robust tumor-targeting ability [124] and low immunogenicity [125]. The content and surface of MSCs-Exo can be covalently or genetically modified [109]. However, this field faces challenges, such as selecting an assured and suitable source of MSCs for delivering therapeutic agents is a serious step; consequently, various MSCs may yield different exosomes varied in size, cargo, and role [115]. Notably, some MSCs-Exo can help tumor development, emphasizing the essential of cautiously characterizing their cargo. Therefore, exosomes of MSCs with tumor-suppressive roles, like umbilical cord-derived-MSCs, are a drug delivery system for cancer therapy [60]. MSCs-Exo must be employed to Good Manufacture Practice (GMP) standards. This field is progressing and requires a profound understanding of exosome kinetics and advances in exosome modifying and loading methods to obtain well cancer treatment. The majority of studies were completed in labs, and the outcomes of clinical application of modified MSCs-Exo remain a problem; this field faces some challenges that are essential to be considered in clinical translation studies. The biology and role of exosomes are not fully revealed. Many questions are associated with exosome biogenesis pathway and uptake, characterizations, nomenclature, and purification, which affect methods and plans that deal with exosome modifying and loading approaches [126]. Mass production of exosomes is another challenge and requirements standardization for their isolation, purification, loading, and modification of exosomes. Mass production of exosomes, especially from MSCs, is very challenging since purification and incubation of human autologous MSCs are laborious and challenging in vitro in a short time. Similar to other exosomes, MSCs-Exo may be captured by the spleen, liver, and lungs following intravenous injection; therefore, exosomes cannot efficiently penetrate the target tissue [127].

## 6. Conclusions

MSCs-Exo are intercellular communication mediators with roles in tumorigenesis and are now the topic of intensive research. A growing body of evidence suggests that MSCs-Exo can support tumor proliferation, metastasis, angiogenesis, immune responses, and drug resistance through different signaling pathways. However, conflicting results have demonstrated that MSCs-Exo may also suppress tumors through several mechanisms. Therefore, the exact role of MSCs-Exo in tumor progression remains controversial; the proposal depends on the source of MSCs, tumor type, and tumor progressive stage. Despite this controversy, it is undeniable that MSCs-Exo has promising potential as a carrier for the delivery of therapeutic agents. MSCs-Exo can be modified on surface and content to improve their tumor-targeting ability. However, the study of MSCs-Exo is still in its infancy, and many problems remain to be dissolved. Clinical translation of MSCs-Exo needs more studies regarding their mass production, isolation, loading, and modification.

## Figures and Tables

**Table 1:** Types of extracellular vesicles.

Extracellular Vesicles	Size	Markers	Mechanism of Generation
Exosomes	30–150 nm	CD9, CD63, CD81, Tsg101	Generated from MVBs through ESCRT-dependent or/and ESCRT-independent mechanism and secreted out of cells upon fusion of MVBs with the plasma membrane
Microvesicles	100–1000 nm	Annexin A1, ARF6	Pocketing from membrane protrusions/the plasma membrane detaching
Apoptotic bodies	1000–6000 nm	Phosphatidylserine	Produced from apoptotic cells

**Table 2:** Promoting function of MSCs-Exo.

MSCs Source	Targeted Tumor	Exosomes Cargo	Function	Ref.
Bone marrow	Multiple myeloma	PSMA3 and PSMA3-AS1	Promote tumor drug resistance	[33]
Mouse bone marrow	Multiple myeloma	Cytokines	Promote the proliferation and migration/drug resistance of tumor cells	[34]
Human bone marrow	Lung cancer	miRNA-21-5p	Promote the growth and migration of tumor cells	[35]
Human bone marrow	Breast cancer	TGF-β, C1q, and semaphorins	Accelerating breast cancer metastasis	[36]
Human bone marrow	Nasopharyngeal carcinoma	FGF19	Induce nasopharyngeal carcinoma growth	[37]
Human umbilical cord	Gastric cancer	-	Promote invasive and EMT ability of tumor cells	[38]
Human umbilical cord	Breast cancer	Platelet-derived growth factor	Promote the angiogenic potential	[39]
Human umbilical cord	Breast cancer	-	Promote the angiogenic potential	[40]
Tumor associated MSCs	Gastric cancer	G6PD-NF-κB-HGF	Promote tumor cell proliferation	[41]
Mouse bone marrow	Breast cancer	miRNA-16	Promote the angiogenic potential	[42]

**Table 3:** Suppression function of MSCs-Exo.

MSCs Source	Targeted Tumor	Exosomes Cargo	Function	Ref.
Human bone marrow	Leukemia	miRNA-223-3p	Suppress leukemia cell proliferation and induce apoptosis	[73]
Human bone marrow	Breast cancer	miRNA-100	Inhibit tumor cell progression	[74]
Human umbilical cord	Endometrial cancer	miRNA-302a	Suppress the proliferation and migration of tumor cells	[75]
Adipose tissue	Hepatocellular carcinoma	miRNA-122	Promote the antihepatocellular carcinoma influence of sorafenib	[76]
Adipose tissue	Hepatocellular carcinoma	miRNA-199a	Increase HCC chemosensitivity	[77]

## Data Availability

Not applicable.
